# Postoperative D-dimer as a signal of venous thromboembolism in patients receiving Edoxaban after hip or knee arthroplasty

**DOI:** 10.1186/s40780-025-00513-7

**Published:** 2025-12-04

**Authors:** Norito Nishiyama, Shiori Iwane, Masayuki Tanaka, Takanori Saito, Toshikazu Tsuji, Noboru Tanigawa

**Affiliations:** 1 Department of Pharmacy, Kansai Medical University Hospital, 2-3-1 Shin- Machi, Hirakata, Osaka 573-1191 Japan; 2 Laboratory of Clinical Pharmacy, Faculty of Pharmaceutical Sciences, Setsunan University, 45-1 Nagaotoge- cho, Hirakata, Osaka 573-0101 Japan; 3 Department of Orthopedic Surgery, Kansai Medical University, 2-5-1 Shin- Machi, Hirakata, Osaka 573-1010 Japan

**Keywords:** D-dimer, Edoxaban, Venous thromboembolism, Total hip arthroplasty, Total knee arthroplasty

## Abstract

**Background:**

Edoxaban is the only direct-acting oral anticoagulant (DOAC) indicated for preventing venous thromboembolism (VTE) in patients undergoing arthroplasty and can be administered for a short period (1–2 weeks). The dosage of edoxaban is set according to renal function and the presence or absence of concomitant medications with P-glycoprotein inhibitory effects. However, a small number of patients still develop VTE after lower-extremity orthopedic surgery (LE-OS), despite edoxaban administration. This single-center, exploratory cohort study retrospectively investigated patients prescribed edoxaban after LE-OS in order to explore the potential of D-dimer as a signal of VTE.

**Methods:**

Of the 1,457 patients who received edoxaban after total hip arthroplasty or total knee arthroplasty between January 2016 and December 2020, 1,244 who cleared the exclusion criteria were divided into the non-VTE group (1,219 patients) and VTE group (25 patients). To reduce bias in VTE occurrence, patient age, sex, body weight, serum creatinine, edoxaban underdosing, previous thromboembolism, bed rest duration ≥ 48 h, and blood transfusion were matched using propensity score matching (PSM). We compared the routinely measured D-dimer values of blood samples collected on postoperative day 7 among non-VTE and VTE groups using the Mann–Whitney U test.

**Results:**

After PSM, there were 23 patients in each of the non-VTE and VTE groups. The median (interquartile range) D-dimer value on postoperative day 7 was 6.2 (4.2–8.1) µg/mL in the non-VTE group and 9.6 (6.3–12.4) µg/mL in the VTE group (*p* = 0.005).

**Conclusions:**

This retrospective exploratory study demonstrated that D-dimer values in patients receiving edoxaban after LE-OS may serve as a signal for VTE occurrence.

## Background

Prevention of venous thromboembolism (VTE), including pulmonary thromboembolism and deep vein thrombosis (DVT), is important after lower-extremity orthopedic surgery (LE-OS) including total hip arthroplasty (THA) and total knee arthroplasty (TKA). VTE occurs in 40–60% of patients who do not receive thromboprophylactic therapy after major orthopedic surgery [1]. The use of anticoagulants for thromboprophylaxis can reduce VTE risk by 50–80% [2, 3].

Edoxaban is the only DOAC indicated for VTE prevention in patients undergoing LE-OS and has two dose options: a standard dose of 30 mg and a lower dose of 15 mg. Although the dosage is regulated according to the patient’s renal function and concomitant medications, some patients who are eligible for the standard dose choose the lower dose (underdosing).

Risk factors for VTE include a previous thromboembolism, prolonged bed rest, aging, malignant tumors, infections, oral contraceptives, estrogen preparations, dehydration, trauma, and fractures [4]. If DVT is suspected, blood tests are performed to check D-dimer levels, followed by imaging studies. Previous studies have reported an association between elevated D-dimer levels and DVT. However, there has been much debate regarding the D-dimer cutoff value. Shiota et al. reported that a D-dimer level of 10 µg/mL on postoperative day 7 had the highest sensitivity and specificity [5]. However, patients in this study were not taking anticoagulants. Anticoagulants have been shown to lower D-dimer levels and reduce diagnostic performance in detecting VTE [6].

This exploratory cohort study retrospectively examined D-dimer levels in patients who received edoxaban after LE-OS, with the aim of exploring its potential as a signal of VTE occurrence.

## Methods

### Patient selection

In this single-center, retrospective cohort study, medical record information of 1,457 inpatients who received edoxaban for VTE prophylaxis after TKA or THA at Kansai Medical University Hospital from January 2016 to December 2020 was collected. The reasons for exclusion comprised: previous edoxaban use for atrial fibrillation (*n* = 4), received higher doses of edoxaban (*n* = 12), unknown edoxaban dose (*n* = 3), unavailable laboratory data (*n* = 110), received their first dose of edoxaban on or after postoperative day 7 (*n* = 5), and received antiplatelet drugs (*n* = 79). A total of 1,244 patients cleared the exclusion criteria and were enrolled.

This study was designed as a retrospective cohort study. Follow-up was initiated on the date of TKA or THA and continued until the occurrence of VTE or the end of the observation period (90 days), whichever came first. Postoperative VTE has a median duration of 16 days, with 77% of cases detected after the first postoperative week and 27% after 4 weeks [7].

From medical records we obtained information such as age, sex, body weight, THA or TKA intervention, medical history, blood transfusion (including autologous blood transfusion), medical prescriptions, serum creatinine, creatinine clearance, and D-dimer levels. Serum creatinine levels were measured at the start of edoxaban administration, while D-dimer levels were measured on postoperative day 7. The orthopedic department routinely measured D-dimer via blood draw on postoperative day 7. All patients had received their first dose of edoxaban by postoperative day 7.

Edoxaban is approved for the prevention of VTE in patients undergoing LE-OS and is generally prescribed at either 30 mg or 15 mg daily. According to Japanese prescribing information, dose reduction to 15 mg is recommended for patients with a creatinine clearance ≤ 50 mL/min, body weight ≤ 60 kg, or concomitant use of P-glycoprotein inhibitors. However, in clinical practice, physicians occasionally choose the 15 mg dose even when the criteria for dose reduction are not strictly met. In this study, patients who received the 15 mg dose without meeting the official criteria were classified as having received an ‘underdosing’. Patients were further divided into two groups: those diagnosed with VTE based on postoperative ultrasound imaging and those without evidence of VTE.

### Statistical analysis

To estimate bias due to confounding factors in the statistical analysis of the VTE and non-VTE patient groups, we employed propensity score matching (PSM) methods. Matching was performed based on age, sex, body weight, serum creatinine, estrogen preparation, edoxaban underdosing, previous thromboembolism, bed rest duration ≥ 48 h, and blood transfusion as covariates. A 1:1 nearest-neighbor matching without replacement was conducted with a caliper width equal to 0.2 of the standard deviation (SD) of the propensity score logit. Consequently, two individuals were excluded using exact or caliper methods. After matching, intergroup balance was assessed by the standardized mean difference (SMD) for each covariate. Generally, a SMD < 0.1 is considered adequate balance, with values up to 0.2 deemed acceptable. All covariates had SMDs < 0.2, indicating a clinically acceptable balance. The continuous variable D-dimer was analyzed via the Mann–Whitney U test. All statistical analyses were performed with EZR (Saitama Medical Center, Jichi Medical University, Saitama, Japan) [8]. A statistically significant difference was considered at a p-value ≤ 0.05.

### Ethical approval

The clinical study protocol was in accordance with the relevant guidelines and regulations, including the Declaration of Helsinki, and was approved by the Ethics Committees of Kansai Medical University (approval number 2021010) and Setsunan University (approval number 2021-021). Given the retrospective nature of this study, both ethics committees waived the need for obtaining informed consent for data use and approved employment of an opt-out procedure for patient consent. The official website of the Kansai Medical University Hospital was used to communicate study information to patients. Authors involved in data analysis did not have access to identifying patient information.

## Results

### Patient characteristics before and after PSM

**Fig. 1:**
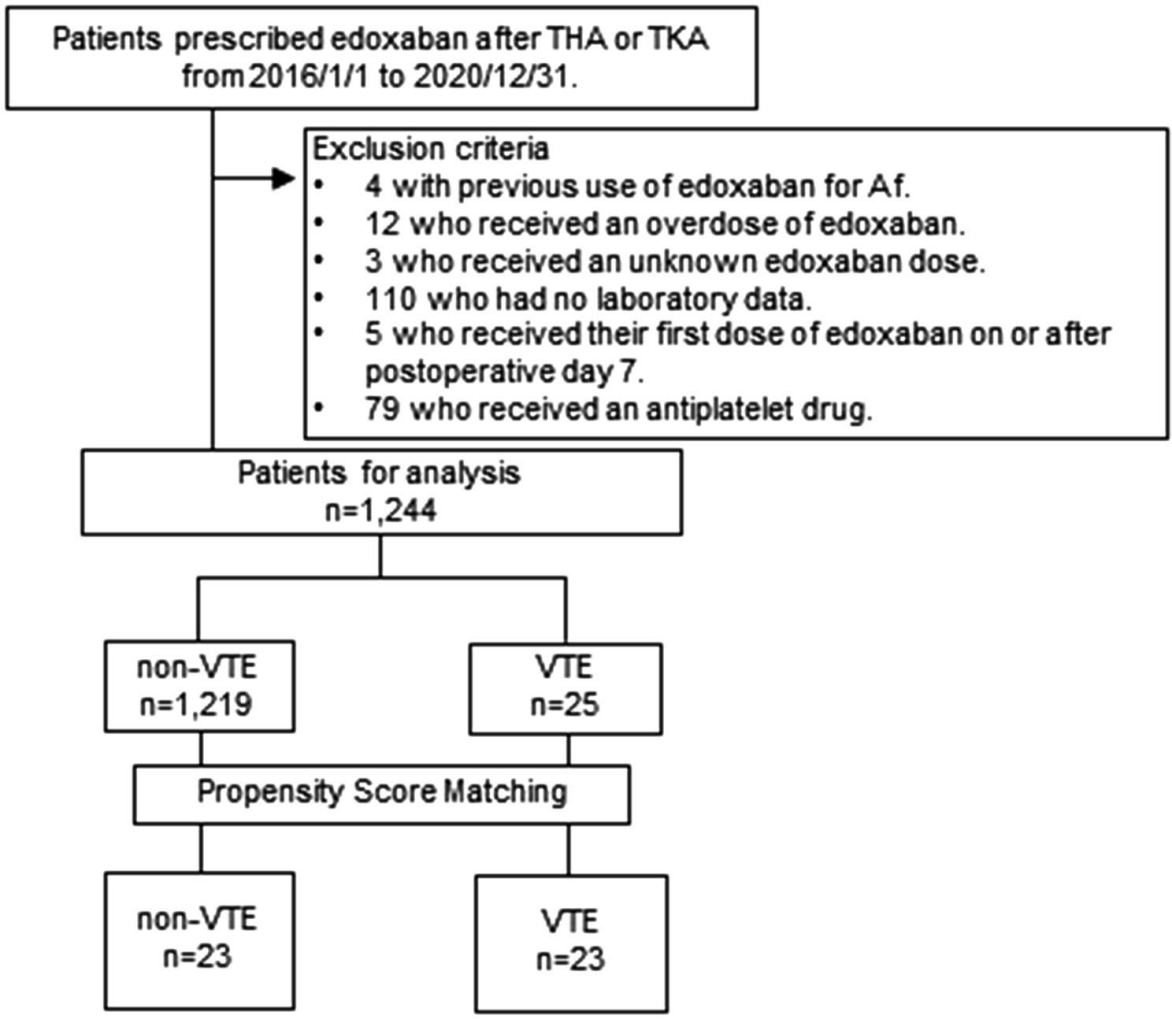
Patient selection flowchart. THA; total hip arthroplasty, TKA; total knee arthroplasty, VTE; venous thromboembolism; Af; atrial fibrillation

In this single-center retrospective study, medical record information of 1,457 inpatients who received edoxaban for VTE prophylaxis after TKA or THA at Kansai Medical University Hospital from January 2016 to December 2020 was collected. The reasons for exclusion comprised: previous edoxaban use for atrial fibrillation (*n* = 4), received higher doses of edoxaban (*n* = 12), unknown edoxaban dose (*n* = 3), and unavailable laboratory data (*n* = 110), received their first dose of edoxaban on or after the seventh postoperative day (*n* = 5), received antiplatelet drugs (*n* = 79). Thus, 1,244 patients who met the inclusion criteria were finally enrolled. Among the 1,457 patients enrolled, 1,244 were administered edoxaban for VTE prevention within 7 days after LE-OS. Of these, 1,219 did not develop a postoperative VTE, while 25 did. PSM was applied to balance the following available patient characteristics: age, sex, body weight, serum creatinine, estrogen preparation, edoxaban underdosing, previous thromboembolism, bed rest duration ≥ 48 h, and blood transfusion. Two patients were excluded because no suitable matches were found. Forty-six patients (*n* = 23, in each group) were ultimately included in the analysis (Fig. 1).

Following PSM, mean age (SD) was 71 (10.9) yrs in the non-VTE group and 72 (7.6) yrs in the VTE group. There were 21 females (91.3%) in each group. Mean body weight (SD) was 65 (20.7) kg in the non-VTE group and 61 (13.1) kg in the VTE group. Mean serum creatinine (SD) was 0.59 (0.17) mg/dL in the non-VTE group and 0.57 (0.14) mg/dL in the VTE group. Since the reference values for serum creatinine differed by sex, mean serum creatinine values were presented separately for females and males. After PSM, there were no patients using estrogen preparations or P-glycoprotein inhibitors. None of the patients had a history of selective estrogen receptor modulator use. PSM produced 14 (60.9%) and 10 (43.5%) cases of THA, and 9 (39.1%) and 13 (56.5%) cases of TKA, in the non-VTE and VTE groups, respectively. The number of patients with edoxaban underdosing, bed rest duration ≥ 48 h, previous thromboembolism, and blood transfusion was matched using PSM. After PSM, the mean (SD) D-dimer value on postoperative day 7 was 6.9 (3.6) µg/mL and 10.5 (5.8) µg/mL in the non-VTE and VTE groups, respectively (Table 1).

**Table 1:** SPatient characteristics before and after PSM

Factor	Include in PSM	Before PSM		After PSM	
		non-VTE (*n* = 1,219)	VTE (*n* = 25)	non-VTE (*n* = 23)	VTE (*n* = 23)
Age, mean (SD)	✓	68 (10.3)	71 (7.7)	71 (10.9)	72 (7.6)
Female, n (%)	✓	1,034 (84.8)	23 (92.0)	21 (91.3)	21 (91.3)
Body weight, mean (SD)	✓	58 (11.7)	61 (12.5)	65 (20.7)	61 (13.1)
**Renal function**					
Scr, overall, mean (SD) (mg/dL)	✓	0.61 (0.17)	0.57 (0.14)	0.59 (0.17)	0.57 (0.14)
Scr, female, mean (SD) (mg/dL)		0.58 (0.14)	0.56 (0.14)	0.57 (0.16)	0.55 (0.14)
Scr, male, mean (SD) (mg/dL)		0.80 (0.20)	0.71 (0.01)	0.86 (0.08)	0.71 (0.01)
**Medical history**					
Estrogen preparation, n (%)	✓	3 (0.2)	0 (0)	0 (0)	0 (0)
P-glycoprotein inhibitors, n (%)		14 (1.1)	0 (0)	0 (0)	0 (0)
**Surgical intervention**					
THA, n (%)		812 (66.6)	11 (44.0)	14 (60.9)	10 (43.5)
TKA, n (%)		407 (33.4)	14 (56.0)	9 (39.1)	13 (56.5)
**Factors affecting VTE**					
Edoxaban underdosing, n (%)	✓	1,116 (91.6)	20 (80.0)	19 (82.6)	19 (82.6)
Bed rest duration ≥ 48 h, n (%)	✓	2 (0.2)	2 (8.0)	0 (0)	0 (0)
Previous thromboembolism, n (%)	✓	18 (1.5)	0 (0)	0 (0)	0 (0)
Blood transfusion, n (%)	✓	126 (10.3)	6 (24.0)	5 (21.7)	6 (26.1)
**Signal for VTE**					
D-dimer on postoperative day 7, mean (SD) (µg/mL)		7.5 (4.0)	10.7 (6.2)	6.9 (3.6)	10.5 (5.8)

Variables used to estimate the PSM are marked with a ✓ in the ‘Include in PSM’ column

PSM; propensity score matching; SD; standard deviation, THA; total hip arthroplasty, TKA; total knee arthroplasty, Scr; serum creatinine, VTE; venous thromboembolism

### D-dimer level on postoperative day 7 as a potential signal of VTE

**Fig. 2:**
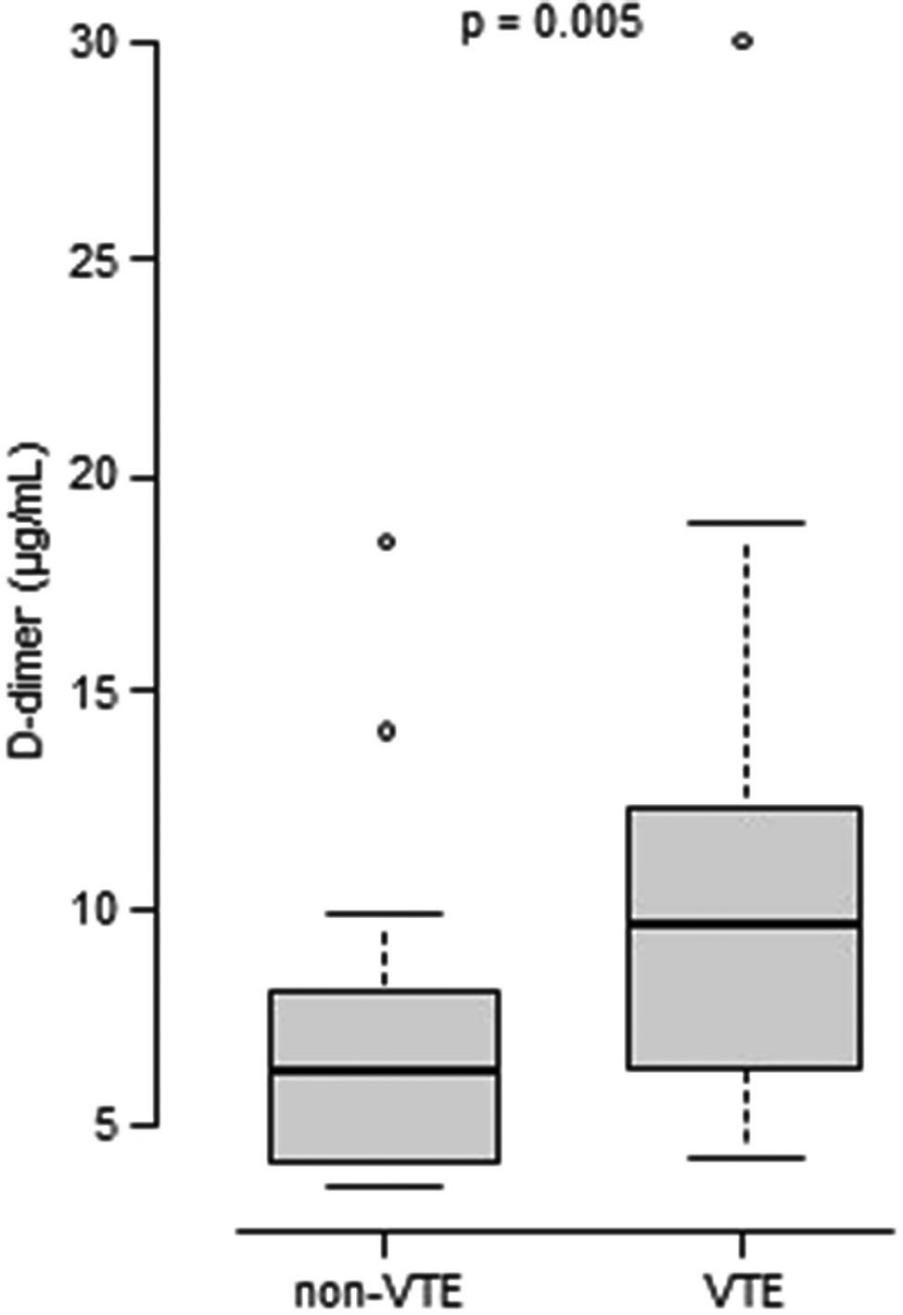
Comparison of D-dimer levels on day 7 after LE-OS between non-VTE and VTE groups. D-dimer values between the non-VTE and VTE groups were compared using the Mann–Whitney U test. The median (interquartile range; IQR) was 6.2 (4.2–8.1) µg/mL in the non-VTE group (*n* = 23) and 9.6 (6.3–12.4) µg/mL in the VTE group (*n* = 23)

D-dimer levels on day 7 after LE-OS were collected for patients receiving edoxaban. After PSM, the median (interquartile range) D-dimer level on postoperative day 7 was 6.2 (4.2–8.1) µg/mL and 9.6 (6.3–12.4) µg/mL in the non-VTE and VTE groups, respectively. A Mann–Whitney U test revealed that this difference was statistically significant (Fig. 2).

## Discussion

In this exploratory analysis of a small, propensity score-matched cohort of patients who underwent LE-OS, those who developed postoperative VTE had higher D-dimer levels, despite edoxaban prophylaxis, than matched patients without VTE. However, given the limited sample size and covariate adjustments, these findings are hypothesis-generating and should not be interpreted as confirmatory or broadly generalizable.

DVT is one of the most common complications in patients undergoing total joint replacement, occurring mostly within 2 weeks, but can appear up to 6 weeks postoperatively [9, 10]. The standard institutional D-dimer cutoff for VTE exclusion is 0.5 µg/mL, although it has been reported that 92% of THA patients and 100% of TKA patients had D-dimer values > 0.4 µg/mL [11]. In this study, the median D-dimer level after TKA or THA was 9.6 µg/mL and 6.2 µg/mL in the VTE and non-VTE groups, respectively, both higher than the threshold. However, anticoagulants can decrease the D-dimer value and reduce its diagnostic potential in VTE detection [12]. We explored the potential of D-dimer as a signal for VTE, focusing exclusively on patients receiving anticoagulant therapy. For inpatients undergoing LE-OS surgery at this hospital, D-dimer measurement on postoperative day 7 was routinely performed.

Antiplatelet agents may be effective at preventing postoperative VTE following LE-OS [13, 14]. Since they are not thought to affect D-dimer levels [15], patients receiving antiplatelet agents were excluded from this analysis. The potential impact of antiplatelet therapy on VTE occurrence could not be evaluated in this study.

Patients undergoing LE-OS are at risk of postoperative infection and receive antibiotics perioperatively. In addition to fever and elevated inflammatory markers, elevated D-dimer levels may also suggest infection [16]. We cannot rule out the possibility that infection and inflammation influenced the D-dimer levels in this study. Essentially, elevated D-dimer levels serve as indicators; when VTE is suspected, clinical symptoms and ultrasound examination are necessary.

This study has several limitations. First, because it was only conducted at a single institution, it carries the inherent possibility of selection bias. The number of observed VTE cases was small, and the number of eligible patients after PSM was substantially reduced from the initial cohort. After PSM, the patients may represent only a specific subset, significantly limiting the external generalizability of conclusions. Second, active malignancy and current smoker status, both established VTE risk factors, were not assessed, introducing the possibility of residual confounding, and either underestimation or overestimation of VTE risk. Third, since the presence of thrombi was not assessed preoperatively in all cases, it was not possible to determine whether thrombi identified postoperatively were directly attributable to surgery. Fourth, the cohort was small and limited to patients post-THA or -TKA. Including surgical type in the PSM disrupted common support between groups, resulting in numerous instances where the control group failed to fall within the predefined caliper. Therefore, to avoid estimation instability stemming from small sample size, surgical type was excluded from the PSM. However, the possibility of residual confounding cannot be ruled out; therefore, the findings should be interpreted conservatively. Additional studies involving larger patient populations are warranted.

## Conclusion

In patients that have undergone LE-OS alongside edoxaban prophylaxis, D-dimer level on postoperative day 7 can be a signal for VTE occurrence.

## Data Availability

The data supporting this study’s findings are available from N.N., S.I., and M.T. upon reasonable request.

## Abbreviations

Scr: Serum creatinine
DVT: Deep vein thrombosis
LE-OS: Lower-extremity orthopedic surgery
PSM: Propensity score matching
THA: Total hip arthroplasty
TKA: Total knee arthroplasty
VTE: Venous thromboembolism
